# The Use of Raman Spectroscopy in the Assessment of the Infiltration Depth of Experimental and Commercial Resin Materials in Extracted Teeth

**DOI:** 10.3390/s26030940

**Published:** 2026-02-01

**Authors:** Andra Clichici, Rareș-Mario Borșa, Anca Onaciu, Nicoleta Simona Vedeanu, Cristina Gasparik, Corina Prodan, Diana Dudea, Mărioara Moldovan, Codruța Saroși, Rareș Ionuț Știufiuc, Valentin Toma

**Affiliations:** 1 Department of Prosthetic Dentistry and Dental Materials, Division of Dental Propaedeutics & Aesthetics, Dental Medicine Faculty, “Iuliu Hatieganu” University of Medicine and Pharmacy, Clinicilor 32, 400001 Cluj-Napoca, Romania; andra.clichici@umfcluj.ro (A.C.); rares.mari.borsa@elearn.umfcluj.ro (R.-M.B.); gasparik.cristina@umfcluj.ro (C.G.); corina.prodan@umfcluj.ro (C.P.); ddudea@umfcluj.ro (D.D.); 2 Department of NanoSciences, Institute of BioMedical Research—MedFUTURE, Louis Pasteur 4-6, 400349 Cluj-Napoca, Romania; anca.onaciu@umfcluj.ro (A.O.); valentin.toma@umfcluj.ro (V.T.); 3 Department of Pharmaceutical Physics & Biophysics, Faculty of Pharmacy, “Iuliu Hatieganu” University of Medicine and Pharmacy, Louis Pasteur 6, 400349 Cluj-Napoca, Romania; simona.vedeanu@umfcluj.ro; 4 Department of Maxillofacial Surgery and Implantology, “Iuliu Hațieganu” University of Medicine and Pharmacy, Cardinal Iuliu Hossu 37, 400029 Cluj-Napoca, Romania; 5 Department of Polymeric Composites, “Raluca Ripan” Chemistry Research Institute, Babes Bolyai University, 400294 Cluj-Napoca, Romania; mmarioara2004@yahoo.com (M.M.); liana.sorosi@ubbcluj.ro (C.S.)

**Keywords:** infiltration depth, Raman spectroscopy, demineralization lesions, infiltration resins

## Abstract

Early enamel lesions result from pH imbalance in the oral cavity, causing subsurface de-mineralization. Resin infiltration has emerged as a minimally invasive treatment option that can halt lesion progression, filling and stabilizing enamel while improving esthetics and microhardness. Raman spectroscopy provides rapid, non-destructive analysis of enamel by detecting molecular vibrations that reflect its chemical composition and structural changes. It allows efficient characterization and depth profiling of dental tissues and materials. Raman spectra also enable quantitative assessment of compositional and structural alterations within enamel. This study aimed to assess the penetration depth of two experimental infiltration materials and a commercial resin within incipient demineralization enamel lesions using Raman spectroscopy. Artificial enamel lesions were created on three extracted human teeth. The samples were treated with a commercial resin infiltrate and two experimental resin infiltrates, with a modified recipe, following the manufacturer’s protocol. Each tooth was sectioned into a 1 mm thick disk. Raman spectra were recorded at sequential depths across both the control (untreated) and infiltrated surfaces of each disk. Characteristic peaks corresponding to infiltrate’s organic matrix and enamel’s phosphate and carbonate groups were employed for assessing penetration depth.

## 1. Introduction

Restorative dentistry is a domain marked by pronounced dynamism, a trend that has become increasingly evident in recent years. Given its close interrelation with the science of dental materials, it is essential that the stringent requirements associated with parameters such as infiltrative capacity, esthetics, mechanical strength, and biocompatibility be subjected to rigorous exploratory characterization, distinguished by high specificity and sensitivity. In this context, ultrasensitive vibrational spectroscopic techniques, along with other analytical methods that are distinct yet complementary to those previously mentioned, may justifiably serve as valuable tools for assessing both the qualitative and quantitative performance of dental materials.

Dental enamel is the hardest tissue in the human body, due to its high content on inorganic material and the highly organized arrangement of this mineral phase [1]. Hydroxyapatite (${Ca}_{10}{{(PO}_{4})}_{10}{\left(OH\right)}_{2}$) represents the highest percentage in dental enamel [2].

The integrity of dental enamel relies on the equilibrium between mineral loss and mineral gain, constantly challenged by factors such as oral hygiene, bacterial metabolism, and diet. A sustained disruption in the dynamic balance between demineralization and remineralization leads to mineral depletion in enamel, making the onset of incipient demineralization lesions possible. This imbalance, generated by acidic pH, determines oral fluids to become undersaturated in phosphate ions. In an attempt to restore ionic balance, hydroxyapatite crystals dissolve them from the enamel [3]. The resulting lesions start as non-cavitated, limited to the outer enamel, with a lower mineral concentration compared to the surrounding sound enamel. Early on, the lesions can be visually identified as white chalky spot lesions. If left unaddressed, they may progress into deeper enamel layers, leading to the loss of hard dental tissues and eventual cavitation [4].

In dentistry, Raman spectroscopy has shown great potential in early recognition of carious lesions as a complementary diagnosis method, evaluating bleaching performance, analyzing the demineralization effect of low pH foods and beverages, and assessing the efficiency of remineralization agents [5,6]. A survey of the contemporary scientific literature reveals a growing interest within the academic community toward advancing research in this direction [7,8].

Raman spectroscopy enables quick detection of chemical composition and structural organization of inorganic tissues. It records the energy profile of light scattered by a laser beam directed onto the sample, generating a unique spectrum based on molecular vibrations. This spectrum allows quantitative analysis and evaluation of structural and biochemical changes. Every Raman spectrum includes a digital fingerprint region (with specific bands of interest), alongside low- and high-frequency regions, where specific bands of interest reflect the molecular and crystalline architecture of the analyte [6].

Demineralization of enamel results in spectral changes, such as a decrease in the intensity of the Raman bands, especially the band corresponding to the phosphate group (${PO}_{4}^{3-}$), not only due to the chemical composition, but also due to the modification of the local orientation of the hydroxyapatite crystals. Thus, the intensity of the phosphate band (960 cm^−1^) is modified [9].

Modern dentistry includes several therapeutic approaches for the incipient demineralization lesions, one of them represented by infiltration resins, which halt the carious progression and mask the enamel opacity. The depth and penetration capacity of the resins represents a key factor in creating a diffusion barrier and therefore the success of the treatment [10]. The demineralization protocol itself exerts a major influence on the infiltration capacity of the evaluated resins. For example, Natarajan et al. investigated the infiltration behavior of several resins using a demineralization protocol based on a mixture of hydrochloric acid, sodium hypochlorite, and hydrogen peroxide. When this protocol was applied to natural carious lesions, the authors reported a markedly higher permeability coefficient compared with specimens subjected to artificially induced demineralization, highlighting the distinct structural characteristics of naturally developed lesions [11].

Other studies have shown varying values for the in-depth infiltration of resin monomers, ranging from ~35 μm to up to almost 180 μm [12]. On the other hand, several studies highlight some inconsistencies regarding the complete obliteration of demineralized enamel by the infiltration resins [10]. The infiltration material penetrates and closes the intercrystalline spaces by creating a polymeric structure that mechanically interlocks the remaining enamel prisms and blocks hydrogen ions, preventing demineralization and advancement of the carious process [13].

Infiltration is effective in filling, strengthening, and stabilizing demineralized enamel. It has the capacity to stop the carious process by increasing enamel microhardness. It also improves dental esthetics. The penetration of resin into the intercrystalline spaces of the enamel is determined by capillary forces influenced by the penetration coefficient [10]. The most of commercial material used for the infiltration of these lesions presents a similar composition and behavior to dental adhesives. As a direct consequence of this, this method has limitations regarding chromatic stability and physicochemical properties. Studies have shown that incorporation of different monomers could overcome the limitations of infiltration resins [14].

Urethane dimethacrylate (UDMA) and 2- hydroxyethyl methacrylate (HEMA) are fundamental constituents of sealants and dental composites. Both are copolymers with a complex structure and a high molecular weight [15]. Their penetration capacity is low, which may limit their ability to reach the full depth of a lesion. The main constituent of the commercially available infiltration material, triethylene glycol dimethacrylate (TEGDMA), endows the material with key characteristics for effective infiltration, namely low viscosity, high polymerization efficiency, and excellent penetration capacity [16]. Due to its low viscosity, the material can infiltrate and occlude enamel microporosities that serve as diffusion pathways for acids, thereby inhibiting the progression of demineralization lesions [10]. TEGDMA has the drawback of low mechanical properties and limited color stability due to its high-water absorption. However, when combined with bisphenol A-glycidyl methacrylate (Bis-GMA) in the resin matrix, it forms a three-dimensional network that enhances both mechanical and chemical characteristics [17].

In this study, Raman spectroscopy was employed to evaluate the penetration capacity of two experimental materials (NB3 and NBF6 from the study of Clichici et al. [18]) and a commercial one on extracted teeth, allowing the assessment of whether the infiltration resins reached the full extent of the artificially induced lesions.

## 2. Materials and Methods

### 2.1. Teeth Included in the Study

Human extracted teeth were used as anonymized specimens, in accordance with the approval of “Iuliu Hațieganu” University of Medicine and Pharmacy Ethics Committee (Approval No. 217/25.01.2023) and in accordance with the provisions of the Declaration of Helsinki. The teeth included in the study were extracted for orthodontic purposes and were included in the present research project based on the written consent provided by the subjects who benefited from this type of dental treatment.

**Figure 1:**
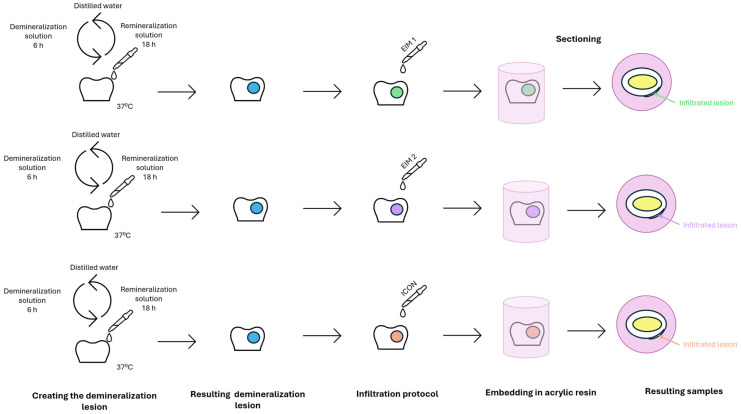
Tooth samples preparation.

As a result of this process, a total of 21 teeth were obtained and further used as biological samples. The following steps of the protocols are illustrated in Figure 1.

### 2.2. Artificially Inducing Incipient Enamel Lesions

Teeth were subjected to a demineralization–remineralization protocol to produce a 6 mm white spot lesion on one surface of each tooth. Teeth were immersed in a demineralization solution (0.075 M acetic acid, 1 mM calcium chloride, and 0.9 mM monopotassium phosphate; pH 4.3) at 37 °C for 6 h, rinsed with distilled water, and then placed in a remineralization solution (150 mM potassium chloride, 1.5 mM calcium nitrate, and 0.9 mM monopotassium phosphate; pH 7) at 37 °C for 18 h. Chemicals required for the preparation of the demineralization and remineralization solutions were obtained by Sigma-Aldrich (Merck, Darmstadt, Germany).

### 2.3. Treatment of the Artificially Induced Incipient Enamel Lesions

The teeth were divided into three equal groups (G1, G2, and G3) and for each group a resin infiltrate was randomly assigned for the treatment of the lesion as follows: G1 was infiltrated with the EIM 1 (70% TEGDMA and 30% UDMA), G2 with the EIM 2 (62% TEGDMA and 14% HEMA, 19% Bis-GMA, and 5% fluorohydroxyapatite and barium fluoride (HAF-${BaF}_{2}$) glass), and group G3 with the commercial material ICON (DMG, Hamburg, Germany). The experimental resins were synthesized at Raluca Ripan Institute of Chemistry Research in Cluj-Napoca. They were previously characterized in terms of mechanical and physicochemical properties, including flexural strength, water absorption and solubility, fluoride release, degree of conversion, residual monomers, and in vitro biocompatibility [18]. The infiltration protocol followed the manufacturer’s instructions for the commercial material, using the etching and drying products provided in the kit.

### 2.4. Preparing the Teeth for Analyses

Each tooth was incorporate in auto-curing acrylic resin (Duracryl; SpofaDental, Jičín, Czech Republic). Afterwards, the teeth were sliced through the middle of the lesion with Extec LabCut 150 cutting machine (Extec Corp., Enfield, CT, USA), with the resulting sliced disks of approximately 1 mm in width. The final teeth samples had a sandwich architecture, presenting a layered structure with the following sequence: encapsulating resin, dental area with the infiltration mixtures, enamel, and dentin. For each tooth, the adjacent untreated side served as the control.

### 2.5. Atomic Force Microscopy Measurements

The typical atomic force microscopy (AFM) experiments have been performed under ambient conditions using an NT-MDT NTegra Vita system (NT-MDT Spectrum Instruments, Zelenograd, Russia) mounted on an inverted Olympus IX73 optical microscope (EvidentScientific, Hamburg, Germany). The measurements have been performed in semi-contact mode using Si_3_N_3_ tips (NT-MDT Spectrum Instruments, Zelenograd, Russia) having a resonant frequency of 235 kHz and a force constant of 12 N/m. The curvature radius of the tips is ~10 nm. The images have been recorded on different regions of the samples in topographic and phase contrast mode.

### 2.6. Raman Measurements

Raman analysis was conducted for each infiltration material, the acrylic resin, and on both the treated and untreated sides of the teeth to obtain their characteristic spectra. For the measurements, a Renishaw inVia Reflex multilaser confocal spectrometer with a resolution of 0.5 cm^−1^ (Renishaw™, Wotton-under-Edge, UK) was used. A NIR laser line (785 nm) was employed for spectral acquisition. The laser power, measured at sample surface, was 113 mW (100% of laser’s output power). The objective used was 50× with a numerical aperture of 0.75. The total signal acquisition time for one measurement was 5 s.

For the infiltration materials and acrylic resin samples, spectral maps consisting of 50 measurements taken at 50 different points were recorded.

In the case of teeth samples, Raman measurements involved linear multi-point mapping to traverse all areas of interest from the encapsulating resin towards the dental tissues, passing through the area affected by infiltration material. A spectral acquisition step size of 0.5 µm was set for the generation of the linear maps. For the adjacent untreated side of the teeth, which served as control, the same experimental setup was applied.

A dedicated Renishaw Wire 4.2 software platform was used for spectra pre-processing in order to eliminate the influence of ambient cosmic radiation, remove technical noise and fluorescence background, and smooth the spectral curves. Finally, the mean spectrum was obtained by averaging the spectra included in the spectral map (OriginPro^®^ 2019).

## 3. Results

### 3.1. Microscopic and Spectroscopic Evaluation of the Intact Tooth

**Figure 2:**
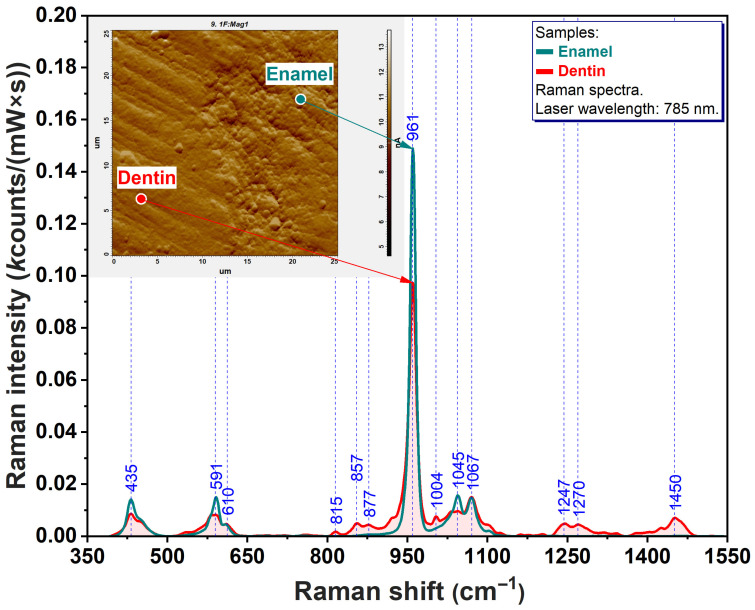
The spectral fingerprint together with the topographic representation of a cross-section through an untreated tooth.

For a better understanding of the infiltrative tropism of the resins under discussion, it was considered appropriate to perform a morpho-compositional analysis of an intact tooth. Thus, atomic force microscopy (AFM) and Raman spectroscopy were employed (Figure 2).

In the inserted AFM image of Figure 2, a cross-section acquired at the crown level of a sound tooth is illustrated. On the right side of this image, a punctate relief can be observed, characteristic of enamel prism morphology. On the opposite side of the image, a series of small, relatively straight, and parallel grooves are visible, corresponding to dentinal tubules. In the central vertical third of the image, a sharply defined boundary with an irregular course (known as the amelo-dentin junction) is noticeable. On the other hand, the Raman spectra of enamel (turquoise) and dentin (red) are presented. Their common feature has dual significance. On the one hand, for both structures, the most intense vibrational band appears at 961 cm^−1^. Last but not least, it is precisely at this spectral position that the most pronounced difference in the spectral morphology of the two tissues becomes evident.

### 3.2. Spectroscopic Assessment of the Individual Infiltration Materials

**Figure 3:**
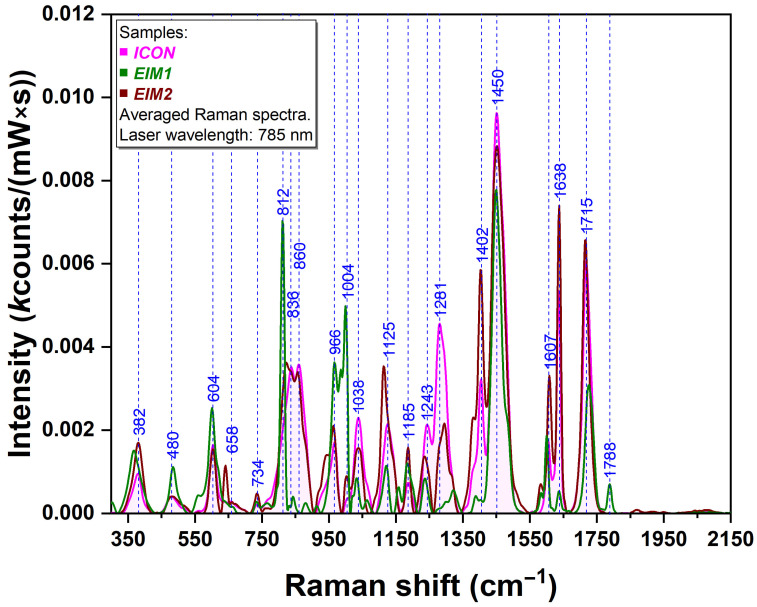
Mean Raman spectra recorded on the infiltration materials (EIM1—green, EIM2—red, and ICON—pink).

Raman spectra were obtained for the following three infiltration materials: ICON (commercial reference) and two experimental formulations, EIM1 and EIM2, with modified compositions. The mean spectra are plotted in Figure 3.

One can notice that some vibrational bands are common for all three materials as follows: 480/485, 602/604, 734, 963/965, 1000/1004, 1033/1038, 1185, 1450, 1601/1607, and 1638 cm^−1^. These similarities confirm the presence of comparable methacrylate monomers and indicate a similar chemical backbone across the three infiltrates. All three spectra are characterized by the dominant nature of the spectral band located at 1450 cm^−1^. At the same time, each material exhibits a series of distinct and specific features, reflecting their compositional variability. Specifically, for EIM1, the most prominent bands are 812, 966, and 1004 cm^−1^. The other experimental resin shows its most intensified spectral bands at 1402, 1638, and 1715 cm^−1^. The commercial resin displays two strongly enhanced bands (1638 and 1715 cm^−1^).

A tentative band assignment for the three materials is presented in Table S1.

### 3.3. Spectroscopic Assessment of Treated and Control Teeth Groups

The purpose of these measurements was to evaluate the penetration depth for each of the three materials and to compare the penetration depth of the two experimental materials, EIM1 and EIM2, with that of the commercial ICON material.

The samples subjected to Raman analysis were evaluated along a linear trajectory covering the different regions characteristic of the materials and the adjacent dental structures. Following a direction from the surface toward the deeper layers, the sequence of regions becomes evident as follows: the acrylic resin area, the enamel zone that underwent acid etching and subsequent treatment with the infiltrative materials, followed further by the underlying dental tissues.

**Figure 4:**
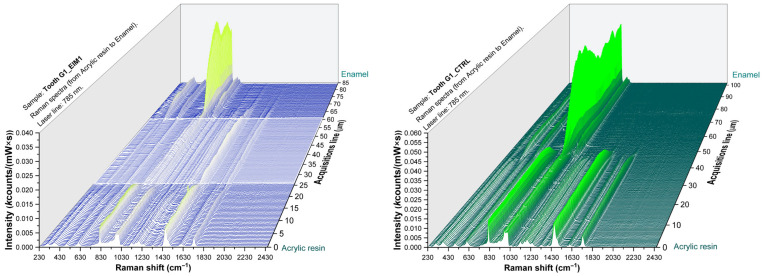
Three-dimensional Raman spectra of a representant tooth from G1 batch treated with EIM1 (**left**) and opposite control side (**right**). The white area illustrates the depth of infiltration of EIM1 resin.

**Figure 5:**
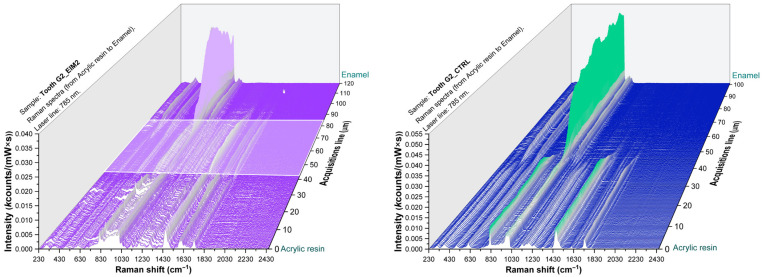
Three-dimensional Raman spectra of a representant tooth from G2 batch treated with EIM2 (**left**) and opposite control side (**right**). The white area illustrates the depth of infiltration of EIM2 resin.

**Figure 6:**
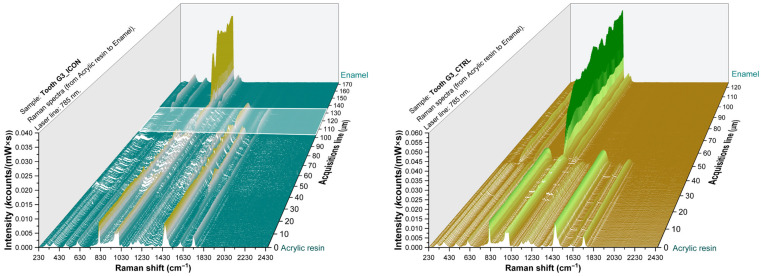
Three-dimensional Raman spectra of a representant tooth from G3 batch treated with ICON (**left**) and opposite control side (**right**). The white area illustrates the depth of infiltration of ICON resin.

Raman spectra of one representative teeth from each group are presented in Figure 4, Figure 5 and Figure 6 illustrating the enamel–resin overlap corresponding to resin infiltration together with the contralateral region used as a control.

The 3D Raman mapping allowed the visualization and quantification of infiltrate’s penetration in the teeth. In order to quantify the mean penetration depth of each resin individually, the twenty-one teeth constituting the three groups included in this study were evaluated. Specifically, for each tooth, the material infiltration depth was assessed based on the obtained Raman maps. These values were then used to compute the group mean and standard deviation (SD) (Table 1).

In the depth-resolved Raman maps, the overlap between enamel and resin spectral features extended over approximately 30 µm for EIM1 and EIM2, whereas ICON showed slightly greater penetration (around 40 µm). This spatial overlap indicates the extent of resin penetration into the sample.

## 4. Discussion

Raman spectroscopy, alongside FT-IR, has proven effective for analyzing the structural and compositional properties of enamel and dentin. By monitoring specific molecular vibrations, such as phosphate bands, the technique can detect changes in mineral content and crystallinity, distinguishing between different tissue states [19].

In the present study, a vibrational spectroscopic analysis was performed to evaluate the infiltration capacity of one commercial resin alongside two experimental resin formulations. One of the main rationales behind initiating this investigation was the need to identify potential alternatives to existing commercial products that may offer improved performance. The experimental resins properties (the influence of monomer composition and filler incorporation on polymerization efficiency, hydrolytic behavior, and cellular response) were evaluated in the study of Clichici et al. [18], support their potential use as infiltration materials for white spot lesions, and provide the basis for the present investigation. To better understand the behavior of the tested resins, it was first necessary to characterize the sound dental substrate. Therefore, a preliminary Raman analysis, complemented by AFM analysis, was conducted on the selected dental tissues. These analyses revealed differences in the intensity of the vibrational band located at 960 cm^−1^, characteristic of the ${PO}_{4}^{3-}$ group present in hydroxyapatite [20]. Such variations reflect the lower degree of mineralization in dentin compared with enamel, a phenomenon directly associated with the differing concentrations of hydroxyapatite in the two tissues.

In a similar manner, Raman analysis was performed on the three infiltrative materials included in the study. The acquired spectra revealed a characteristic spectral fingerprint for each resin. Although all three materials exhibited, in most cases, a set of common vibrational bands, these bands differed in intensity. This observation indicates a shared chemical homogeneity among the constituent components of the resins, with the differences arising solely from the relative proportions of these components. Raman analysis revealed that all three materials exhibit a vibrational band at 1450 cm^−1^, corresponding to the scissoring mode of CH_2_/CH_3_ groups [8] typically found in resins. This observation indicates that the materials share a similar chemical structure. The relative intensities of the Raman bands differ among the materials, which may reflect variations in penetration depth or chemical composition.

In the process of evaluating the sections subjected to Raman analysis, the three-dimensional spectra analysis allows the identification of a region in which both the molecular signatures characteristic of each individual layer and the transitional interfacial zones can be clearly distinguished. This approach allows a nanometric-scale assessment of the infiltration distances of the studied materials.

The penetration depths observed for the three tested materials indicate comparable infiltration capacities within the enamel. EIM 1 infiltrated ~30 µm, while EIM 2 reached around 32 µm, suggesting that both experimental resins perform similarly in terms of enamel penetration. The commercial ICON resin exhibited slightly greater infiltration, up to 40 µm, which may reflect differences in resin composition, viscosity, or interaction with the enamel matrix.

The difference in penetration depth between the commercial resin (42 µm) and the experimental formulations (~30–32 µm) can be attributed to the rheological properties of the monomers. Commercial resins optimized for infiltration, such as Icon, contain a higher proportion of low-viscosity monomers like TEGDMA (0.01 Pa·s, 286 g/mol), which enhances molecular mobility and facilitates rapid penetration into porous structures. In contrast, experimental resins rich in Bis-GMA (1200 Pa·s) and UDMA (23 Pa·s) exhibit higher viscosities and stronger intermolecular interactions, including hydrogen bonding, which act as physical barriers and limit diffusion. These differences explain the lower infiltration depth observed for the experimental materials and highlight the key role of monomer flexibility and viscosity in determining penetration efficiency [21].

The identification of material-specific vibrational bands (EIM 1: 1450, 1725 cm^−1^; EIM 2: 1450, 1638, 1715 cm^−1^; and ICON: 1450, 1750 cm^−1^) and their clear distinction from enamel bands (435, 591, 961, and 1067 cm^−1^) provides a reliable method to map resin infiltration and confirm that observed spectral features correspond exclusively to the restorative material.

These results suggest that both experimental resins have infiltration potential comparable to commercial products, supporting the possibility of developing alternative formulations. Small differences in penetration depth could be further investigated in relation to resin composition, polymerization kinetics, or long-term stability.

Furthermore, several vibrational bands specific exclusively to dental tissues (435 cm^−1^—out of plane bending of ${PO}_{4}^{3-}$, 591 cm^−1^—in plane bending of ${PO}_{4}^{3-}$ [22], and 961 cm^−1^—symmetric stretching of ${PO}_{4}^{3-}$ [20]) served as reliable markers for the topographical delineation of enamel regions infiltrated solely by the restorative infiltrates. This facilitated a precise evaluation of infiltration depth.

The spectral overlap between enamel and resin signals in the infiltrated regions indicates efficient infiltration for all tested materials. Raman spectra collected from the infiltrated enamel surfaces revealed the coexistence of mineral (hydroxiapatite ${PO}_{4}^{3-}$, ~960 cm^−1^) [20] and organic resin bands (1450–1720 cm^−1^) [8,23,24,25], confirming resin penetration into the porous enamel structure. In contrast, spectra from control (untreated) regions showed only the phosphate signal of hydroxyapatite, without organic features. A depth profile obtained across the resin–enamel interface shows a gradual decrease in the intensity of organic bands toward the inner enamel. This pattern reflects limited penetration depth, consistent with the microporous nature of early enamel lesions. The overlapping of resin and enamel signals in the infiltrated zones confirms that all tested materials were capable of diffusing into the enamel lesion area, with negligible interference from the embedding medium.

In contrast, the spectra from the control regions reflect pure enamel, devoid of resin or embedding material. This confirms that the embedding medium did not interfere with the tooth substrate or the infiltration zones.

Overall, the spectral analysis confirms that Raman spectroscopy is a robust tool for evaluating the distribution and depth of resin infiltration in enamel lesions.

## 5. Conclusions

The results obtained in this pilot study reflect the applicative potential of restorative materials that may serve as alternatives to currently available commercial products. Without establishing a direct comparison between the experimental resins and the commercial material, the findings nonetheless indicate that all three materials exhibit nearly similar infiltration capacities. Despite the promising outcomes, further investigations are imperative, including studies with larger sample sizes, optimization of the experimental resin formulations, and long-term evaluation of their mechanical and esthetic properties. These findings may also open new research avenues in this fascinating field of materials with biomedical applications.

## Figures and Tables

**Table 1:** Infiltrate’s penetration depth.

Material	Average Distance ± SD (µm)
EIM1	30.8 ± 16.8
EIM2	32.3 ± 19.0
Icon	42 ± 18.6

## Data Availability

Data is contained within the article or Supplementary Materials.

## Supplementary Materials

The following supporting information can be downloaded at https://www.mdpi.com/article/10.3390/s26030940/s1, Table S1: The most representative wavenumbers and associated molecular groups. References [26,27,28,29,30] are cited in the Supplementary Materials.

## Abbreviations

The following abbreviations are used in this manuscript:

EIM	Experimental infiltration material
UDMA	Urethane dimethacrylate
HEMA	2- hydroxyethyl methacrylate
TEGDMA	Triethylene glycol dimethacrylate
Bis-GMA	Bisphenol A-glycidyl methacrylate
AFM	Atomic force microscopy
