# Supplementary material for: The Use of Raman Spectroscopy in the Assessment of the Infiltration Depth of Experimental and Commercial Resin Materials in Extracted Teeth
Source: Sensors (Basel). 2026 Feb 1;26(3):940. doi: 10.3390/s26030940 (PMC12899240; doi:10.3390/s26030940)
Supplement: Supplementary file 1 [file sensors-26-00940-s001.zip › sensors-4050779-supplementary.pdf]

Supplementary files

The Use of Raman Spectroscopy in the Assessment of the Infiltration Depth of Experimental and Commercial Resin Materials in Extracted Teeth

Andra Clichici [1,†], Rareș-Mario Borșa [1,2,3,4,†], Anca Onaciu [2,†], Nicoleta Simona Vedeanu [3], Cristina Gasparik [1], Corina Prodan [1], Diana Dudea [1], Mărioara Moldovan [5], Codruța Saroși [5], Rareș Ionuț Știufiuc [2,3,*] and Valentin Toma [2]

1 Department of Prosthetic Dentistry and Dental Materials, Division Dental Propaedeutics & Aesthetics, Dental Medicine Faculty, "Iuliu Hatieganu" University of Medicine and Pharmacy, Clinicilor 32, 400001 Cluj-Napoca, Romania; andra.clichici@umfcluj.ro (A.C.); rares.mari.borsa@elearn.umfcluj.ro (R.M.B.); gasparik.cristina@umfcluj.ro (C.G.); corina.prodan@umfcluj.ro (C.P.); ddudea@umfcluj.ro (D.D.)
2 Department of NanoSciences, Institute of BioMedical Research—MedFUTURE, Louis Pasteur 4-6, 400349 Cluj-Napoca, Romania; anca.onaciu@umfcluj.ro (A.O.); valentin.toma@umfcluj.ro (V.T.)
3 Department of Pharmaceutical Physics & Biophysics, Faculty of Pharmacy, "Iuliu Hatieganu" University of Medicine and Pharmacy, Louis Pasteur 6, 400349 Cluj-Napoca, Romania; simona.vedeanu@umfcluj.ro
4 Department of Maxillofacial Surgery and Implantology, "Iuliu Hațieganu" University of Medicine and Pharmacy, Cardinal Iuliu Hossu 37, 400029 Cluj-Napoca, Romania
5 Department of Polymeric Composites, "Raluca Ripan" Chemistry Research Institute, Babes Bolyai University, 400294 Cluj-Napoca, Romania; mmarioara2004@yahoo.com (M.M.); liana.sorosi@ubbcluj.ro (C.S.)
* Correspondence: rares.stiufiuc@umfcluj.ro
† These authors contributed equally to this work.

Table S1. The most representative wavenumbers and associated molecular groups

EIM 1	EIM 2	ICON	Assignment	Reference
	382	382	dicalcium phosphate dihydrate	[1]
602	604	604	ν_3 (PO_4^{3-}) in hydroxiapatite	[2]
	642		ν(C–COO) in hydroxiapatite	[3]
734	734	734	ν (C–C) skeletal mode	[4]
764			ν_4 (CO_3^{2-}) in hydroxiapatite	[5]
812			ν (CC) skeletal backbone	[6]
834		836	ν(CH_2)	[4]
	858	860	δ(CCH) aromatic, ν (CC)	[6]
880			dicalcium phosphate dihydrate	[1]
963	965	966	ν_1 (PO_4^{3-}) in hydroxiapatite	[2]
1000	1003	1004	ν (CC) aromatic ring breathing, ν_1(HPO_4^{2-})	[2]
1033	1033	1038	ν_3 (PO_4^{3-})	[2]
	1114		νa(C–O–C) and C–C skeleton backbone in hydroxiapatite	[3]
1185	1185	1185	νa(C–O–C) and C–C skeleton backbone	[3]
1237	1237	1243	ν(C–O), ν (C–COO)	[4]
	1402	1402	ν (C=CH_2)	[8]
1450	1450	1450	δ(CH_2)	

				[8,9]
1607			ν (C-H)	[10]
	1607	1607	Phenyl ring	[11]
1638	1638	1638	ν (C=C)	[11]
	1715	1715	ν (C=O)	[12]
1725	1725	1725	ν (C=O)	[11]

ν_1 - symmetric stretching; ν_2 -out-of-plane bending; ν_3 - asymmetric stretching; ν_4 - in-plane bending; ν– stretching; δ - scissoring

References

[1] Seredin, P.; Goloshchapov, D.; Prutskij, T.; Ippolitov, Y. Phase Transformations in a Human Tooth Tissue at the Initial Stage of Caries. *PLoS One*, **2015**, *10*, e0124008.

[2] Penel, G.; Leroy, G.; Rey, C.; Bres, E. MicroRaman Spectral Study of the PO 4 and CO 3 Vibrational Modes in Synthetic and Biological Apatites. *Calcif. Tissue Int.*, **1998**, *63*, 475–481.

[3] Iordache, S.-M.; Iordache, A.-M.; Gatin, D.I.; Grigorescu, C.E.A.; Ilici, R.R.; Luculescu, C.-R.; Gatin, E. Performance Assessment of Three Similar Dental Restorative Composite Materials via Raman Spectroscopy Supported by Complementary Methods Such as Hardness and Density Measurements. *Polymers (Basel).*, **2024**, *16*, 466.

[4] Xingsheng, X.; Hai, M.; Qijing, Z.; Yunsheng, Z. Properties of Raman Spectra and Laser-Induced Birefringence in Polymethyl Methacrylate Optical Fibres. *J. Opt. A Pure Appl. Opt.*, **2002**, *4*, 237–242.

[5] Awonusi, A.; Morris, M.D.; Tecklenburg, M.M.J. Carbonate Assignment and Calibration in the Raman Spectrum of Apatite. *Calcif. Tissue Int.*, **2007**, *81*, 46–52.

[6] Natarajan, A.K.; Fraser, S.J.; Swain, M. V.; Drummond, B.K.; Gordon, K.C. Raman Spectroscopic Characterisation of Resin-Infiltrated Hypomineralised Enamel. *Anal. Bioanal. Chem.*, **2015**, *407*, 5661–5671.

[7] Rath, S. Raman Spectroscopy as Spectral Tool for Assessing the Degree of Conversion after Curing of Two Resin-Based Materials Used in Restorative Dentistry. *Adv. Mater. Sci. Res.*, **2022**, *5*, 66–69.

[8] Gatin, E.; Iordache, S.-M.; Matei, E.; Luculescu, C.-R.; Iordache, A.-M.; Grigorescu, C.; Ilici, R. Raman Spectroscopy as Spectral Tool for Assessing the Degree of Conversion after Curing of Two Resin-Based Materials Used in Restorative Dentistry. *Diagnostics*, **2022**, *12*, 1993.

[9] Kirchner, M.T.; Edwards, H.G.M.; Lucy, D.; Pollard, A.M. Ancient and Modern Specimens of Human Teeth: A Fourier Transform Raman Spectroscopic Study. *J. Raman Spectrosc.*, **1997**, *28*, 171–178.

[10] Par, M.; Gamulin, O.; Marovic, D.; Klaric, E.; Tarle, Z. Raman Spectroscopic Assessment of Degree of Conversion of Bulk-Fill Resin Composites – Changes at 24 Hours Post Cure. *Oper. Dent.*, **2015**, *40*, E92–E101.

[11] Shin, W.S.; Li, X.F.; Schwartz, B.; Wunder, S.L.; Baran, G.R. Determination of the Degree of Cure of Dental Resins Using Raman and FT-Raman Spectroscopy. *Dent. Mater.*, **1993**, *9*, 317–324.

[12] Soares, L.E.S.; Rocha, R.; Martin, A.A.; Pinheiro, A.L.B.; Zampieri, M. Monomer Conversion of Composite Dental Resins Photoactivated by a Halogen Lamp and a LED: A FT-Raman Spectroscopy Study. *Quim. Nova*, **2005**, *28*, 229–232.
